# Point-of-admission hypothermia among high-risk Nigerian newborns

**DOI:** 10.1186/1471-2431-8-40

**Published:** 2008-10-06

**Authors:** Tinuade A Ogunlesi, Olusoga B Ogunfowora, Folashade A Adekanmbi, Bolanle M Fetuga, Durotoye M Olanrewaju

**Affiliations:** 1 Department of Paediatrics, College of Health Sciences, Olabisi Onabanjo University, Sagamu, Nigeria

## Abstract

**Background:**

Facilities which manage high-risk babies should frequently assess the burden of hypothermia and strive to reduce the incidence.

**Objective:**

To determine the incidence and outcome of point-of-admission hypothermia among hospitalized babies.

**Methods:**

The axillary temperatures of consecutive admissions into a Nigerian Newborn Unit were recorded. Temperature <36.5°C defined hypothermia. The biodata and outcome of these babies were studied.

**Results:**

Of 150 babies aged 0 to 648 hours, 93 had hypothermia with an incidence of 62%. Mild and moderate hypothermia accounted for 47.3% and 52.7% respectively. The incidence of hypothermia was highest (72.4%) among babies aged less than 24 hours. It was also higher among out-born babies compared to in-born babies (64.4% *vs *58.3%). Preterm babies had significantly higher incidence of hypothermia (82.5%) compared with 54.5% of term babies (RR = 1.51; CI = 1.21 – 1.89). The incidence of hypothermia was also highest (93.3%) among very-low-birth-weight babies.

The Case-Fatality-Rate was significantly higher among hypothermic babies (37.6% vs 16.7%; RR = 2.26, CI = 1.14 – 4.48) and among out-born hypothermic babies (50% vs 17.1%; RR = 0.34, CI = 0.16 – 0.74). CFR was highest among hypothermic babies with severe respiratory distress, sepsis, preterm birth and asphyxia.

**Conclusion:**

The high incidence and poor outcome of hypothermia among high-risk babies is important. The use of the 'warm chain' and skin-to-skin contact between mother and her infant into routine delivery services in health facilities and at home may be useful.

## Background

Neonatal hypothermia, defined by the World Health Organization (WHO) as axillary temperature less than 36.5°C, [1] is a major contributor to neonatal illnesses and deaths both in the developed and developing parts of the world. [2-5]

The body temperature of a newborn infant tends to fall progressively after birth as part of the transition form the intra-uterine to extra-uterine environment [1] particularly among babies who are preterm, low birth weight or sick infants. [6] In most parts of the developing world, including Nigeria, where these groups of babies are commonly encountered in clinical practice, [4] it is important to regularly assess the burden of neonatal hypothermia in order to highlight the need for improved preventive interventions.

There appears to be a lot of variations in the definition of this important condition. Several workers in different parts of the world have studied neonatal hypothermia using different definitions based on different sites of temperature measurement and different values. [7-9] This may explain the myriad of conflicting reports on the incidence of this condition in different newborn centres worldwide. Therefore, the definition and method of classification of neonatal hypothermia as recommended by the WHO [1] provides standardization for researchers in newborn care.

This study is aimed at determining the incidence and outcome of point-of-admission hypothermia among Nigerian high-risk babies using the WHO definition and protocol. [1] This study is done with a view to developing a more proactive management approach that may reduce the occurrence of neonatal hypothermia.

## Methods

This cross-sectional survey was done at the Neonatal Unit of the Olabisi Onabanjo University Teaching Hospital, Sagamu, Nigeria between July 2007 and January 2008. The unit provides specialist neonatal care for babies delivered in the maternity unit of the hospital and those referred to the hospital from other health facilities in and around Sagamu. Ethical approval was obtained form the Scientific Review and Ethical Committee of the hospital. Verbal consent was also obtained from the parent or caregiver of each baby.

Axillary temperature for all consecutive admissions was measured with a digital thermometer (MT-101 model of UMEC^®^) which could measure from 32°C to 42.9°C with an accuracy of ± 0.2°C at the point of admission using standard procedure. [1] Using the WHO classification, axillary temperature between 36.5°C and 37.5°C was recorded as normal while temperature less than 36.5°C was recorded as hypothermic. [1] Hypothermia was further classified into mild (36°C to 36.4°C), moderate (32°C to 35.9°C) and severe (<32°C). Other data obtained from the subjects included the clinical diagnoses, age in hours, sex, body weight, estimated gestational age (EGA) and the places of delivery like General Hospitals, Primary Health Centres and Private Clinics (Health Facilities), Traditional Birth Centres and homes. The outcome of hospitalization was recorded as discharge, death and discharge against medical advice for each baby. Post-mortem examinations were not routinely done for reasons of socio-cultural disapproval.

The data were analysed with SPSS 15.0 version using the Student's t-test, Risk Ratio (RR) and the 95% Confidence Interval (CI). Statistical significance was established when p values were less than 0.05 or the 95%CI did not include unity.

## Results

A total of 150 were studied. These comprised 60 (40%) in-born babies and 90 (60%) out-born babies. The out-born babies were delivered in health facilities (59; 65.6%), homes (17; 18.8%) and traditional birth centres (14; 15.6%). These babies were aged 0 to 648 hours with the mean of 67.4 ± 121.4 hours. Majority of the babies (87; 58%) presented within the first 24 hours of life while the remaining 24 (16%), 22 (14.7%) and 17 (11.3%) presented between 24 to 72 hours, 73 to 168 hours and more than 168 hours of age respectively.

There were 93 (62%) males and 57 (38%) females. The estimated gestational age ranged from 28 to 41 weeks with the mean of 37.3 ± 2.9 weeks while the body weight varied between 1 kg and 5 kg with the mean of 2.6 ± 0.7 kg. Forty (26.7) babies were preterm while 87 (58%) were small-for-gestational age (SGA).

### Pattern of Body Temperature

The body temperature ranged from 33.3°C to 39.4°C with the mean of 36.2 ± 1.0°C. Forty eight (32%) babies had normal body temperature while the remaining 93 (62%) and 9 (6%) had hypothermia and hyperthermia respectively. Of the 93 hypothermic babies, 44 (47.3%) and 49 (52.7%) had mild and moderate hypothermia respectively.

### Comparison of the clinical parameters of babies with and without hypothermia

The mean age of 93 hypothermic babies was 48.8 ± 110.5 hours compared with 71.9 ± 109.8 hours for the 48 babies with normal body temperature without statistical significance (t = 1.18, p = 0.241). However, the mean EGA of babies with hypothermia was significantly lower compared with that of babies with normal temperature (36.7 ± 3.2 weeks *vs *38.1 ± 2.5 weeks; t = 3.0, p = 0.003). Similarly, babies with hypothermia had significantly lower mean body weight compared with babies with normal body temperature (2.4 ± 0.8 kg *vs *2.8 ± 0.6 kg; t = 3.05, p = 0.003).

### Incidence of hypothermia

The incidence of hypothermia in this study was 62% (93/150). The incidence of early hypothermia (occurring within the first 72 hours of life) was 67.6% (75/111) compared with 46.2% (18/39) for late hypothermia (occurring after the first 72 hours of life). The difference in incidence was statistically significant (RR = 1.46; CI = 1.02 – 2.10). Thirty five (58.3%) in-born babies were hypothermic compared with 58 (64.4%) out-born babies but the difference in incidence was not statistically significant (RR = 0.90; CI = 0.70 – 1.18).

Table 1 shows that the incidence of hypothermia was highest (72.4%) among babies aged less than 24 hours while it was lowest among babies aged more than 168 hours (35.3%). The incidence of hypothermia was 71.7% (33/46), 94.7% (18/19), 58.3% (7/12) and 50.0% (5/10) among babies aged 1 hour or less, 2 to 4 hours, 5 to 12 hours and 13 to 24 hours respectively. Hypothermia also occurred more frequently among out-born babies aged 24 hours or less than in-born babies of similar age (30/33 (90.9%) vs 33/54 (61.1%); RR = 1.49, CI = 1.17 – 1.89) whereas the incidence was similar among in-born and out-born babies aged more than 24 hours (33.3% *vs *49.1%; RR = 0.68, CI = 0.21 – 2.17).

**Table 1:** Distribution of subjects according to age and body temperature

Age (Hours)	Total	Hypothermia	Normothermia	Hyperthermia
<24	87	63 (72.4)	24 (27.6)	0 (0.0)
24 – 72	24	12 (50.0)	10 (41.7)	2 (8.3)
73 – 168	22	12 (54.5)	8 (36.4)	2 (9.1)
>168	17	6 (35.3)	6 (35.3)	5 (29.4)
Total	150	93	48	9

KEY: Figures in parentheses are percentages of the total in each row.

The incidence of hypothermia decreased steadily with increasing body weight; it was 93.3% (14/15) for babies < 1.5 kg, 84.3% (43/51) for babies weighing between 1.5 and 2.49 kg and 42.9% (36/84) for babies who weighed 2.5 kg or more. Thirty three (82.5%) of the preterm babies had hypothermia compared with 60 (54.5%) of term babies with statistical significance (RR = 1.51; CI = 1.21–1.89). However, incidence of hypothermia was insignificantly higher among SGA babies (57; 65.5%) compared with babies who were appropriate and large-for-gestational age (36; 57.1%) (RR = 1.15; CI = 0.88 – 1.49).

The incidence of moderate hypothermia was also insignificantly higher among out-born babies compared with in-born babies (60.3% *vs *40.0%; RR = 1.50; CI = 0.96 – 2.38).

### Outcome

Fifty-four (58.1%), 35 (37.6%) and 4 (4.3%) hypothermic babies were discharged home well, died or were discharged against medical advice respectively. Similarly, 36 (75.0%), 8 (16.7%) and 4 (8.3%) of babies with normothermia were discharged home, died or were discharged against medical advice. Eight (22.9%) of the hypothermic fatalities occurred within 24 hours of admission whereas none of the normothermic deaths occurred within 24 hours of admission.

Although, the Case Fatality Rate (CFR) for hypothermic babies (37.6%) was significantly higher than 16.7% for babies with normal body temperature (RR = 2.26; CI = 1.14 – 4.48), their mean durations of hospitalization were similar (10.6 ± 9.6 days *vs *9.6 ± 7.4 days; t = 0.64, p = 0.5).

The CFR among hypothermic babies was highest in babies who weighed < 1 kg (66.7%) and lowest among babies who weighed at least 2.5 kg. It was 36.4% and 39.5% among babies who weighed 1 – 1.49 kg and 1.5 – 2.49 kg respectively.

The CFR among in-born hypothermic babies was significantly lower than that of outborn hypothermic babies (17.1% *vs *50%; RR = 0.34, CI = 0.16 – 0.74). Similarly, CFR among hypothermic babies aged 24 hours or less was significantly lower among inborn babies compared to out-born babies (15.2% *vs *55.6%; RR = 0.28, CI = 0.12 – 0.68) whereas the CFR among hypothermic babies aged more than 24 hours was not significantly different among in-born and out-born babies (50.0% *vs *45.2%; RR = 1.07, CI = 0.25 – 4.55).

Twenty three (46.9%) out of 49 babies with moderate hypothermia compared with 12 (27.3%) out of 44 babies with mild hypothermia died but this difference was not statistically significant (RR = 1.72; 0.98 – 3.03). The babies with moderate hypothermia also had an insignificantly longer duration of admission compared with babies with babies with mild hypothermia (11.3 ± 11.2 days *vs *9.7 ± 7.5 days; t = 0.8, p = 0.426).

Table 2 describes the CFR among hypothermic babies with specific clinical diagnoses. The CFR was highest among hypothermic babies with severe respiratory distress, preterm birth, sepsis and asphyxia. However, no fatality was recorded among hypothermic babies with jaundice, bleeding disorders, aspiration syndromes and congenital malformations.

**Table 2:** Case Fatality Rates in hypothermic babies with specific clinical disorders

**Clinical disorders**	**Number of hypothermic babies**	**Number of deaths (CFR)**
Asphyxia	58	22 (37.9)
Preterm	32	14 (43.8)
Jaundice	15	0 (0.0)
Sepsis	14	6 (42.9)
Severe respiratory distress	10	7 (70.0)
Bleeding disorders	2	0 (0.0)
Aspiration syndromes	4	0 (0.0)
Congenital malformation	4	0 (0.0)

**CFR **– Case Fatality Rate (expressed as percentage)

Some babies had multiple diagnoses.

## Discussion

The incidence of hypothermia in this study (62%) is higher than 44% previously reported among Zambian babies,[9] 22.4% among Tanzanian babies [10] but lower than 85% reported among Zimbabwean babies.[11] These studies are comparable because they were conducted on the African continent and their definition of hypothermia was uniform. The varying incidence of hypothermia may be due to differences in patients' selection. Nevertheless, the high frequency of neonatal hypothermia obtained in this study is a matter of concern thus justifying the study.

Therefore, more attention needs to be focused on preventive measures.

The incidence of hypothermia was highest in the first 24 hours of life and among preterm and low-birth-weight babies in this study. This observation agrees with previous reports. [6,12] This group of babies typically have poor subcutaneous tissue and immature central thermoregulatory functions. [13] Hypothermia had been reported to occur most frequently within the first one hour of life even among in-born babies [12] but that was not our observation in this study. The observation in this study may reflect the fact that most of the admissions within the first 24 hours of life were due to perinatal problems related to labour and delivery. Such critical newborn sicknesses have been shown to cause hypothermia. [1] Therefore, critically ill babies deserve additional thermal management apart from the routine.

While it is not unusual that out-born deliveries are significantly hypothermic, [10,14] the similarly high incidence of hypothermia among in-born babies is difficult to explain since routine hospital care of newborn babies also includes the use of the 'warm chain'. [1] However, most hospitalized in-born babies are high-risk and often critically ill. Such critical illness associated with hypothermia may include perinatal asphyxia, infections and respiratory distress. [15] Therefore, the body temperature in babies with these conditions should be monitored more frequently than the routine for early diagnosis of hypothermia and prompt institution of thermal management.

Most centres in the developing world lack efficient warming devices. [1] This also applies to our centre where efficient incubators and other warming devices like radiant heater are few. Therefore, skin-to-skin contact which has been reported to be efficient in the prevention and treatment of hypothermia particularly among preterm infants [16] should be incorporated into the routine care of newborn babies particularly, the preterm and low-birth-weight. Specifically, routine hospital care of newborns should also include skin-to-skin contact with the mother (when a baby is positioned on the mother's bare abdomen and chest to provide warmth from the heat generated by the mother's body) since it has been shown that babies that are kept close to their mothers achieved better body temperature control than those nursed in cots away from the mothers. [17]

It is equally significant that more than a third (37.6%) of the hypothermic infants in this study died. This is remarkably higher than 18.3% reported among Zimbabwean babies, [11]. The relatively higher CFR among hypothermic babies agrees with previous reports [10] and it could be attributed to the contribution of out-born hypothermic babies. These babies also had significantly higher CFR compared with the in-born hypothermic babies particularly within the first 24 hours of life. Hypothermia is known to be associated with deaths among referred babies. [14] Thus, it is not surprising that the CFR was significantly higher among babies delivered outside our hospital as previously observed. [11] This may be due to the tendency to be more ill than in-born babies in addition to poor transportation techniques and delayed commencement of appropriate therapeutic interventions. The contribution of out-born babies to both the high incidence and poor outcome associated with neonatal hypothermia may necessitate a community-oriented programme that would introduce the 'warm chain' [1] to health workers who care for newborn babies at the primary and secondary levels of health care as well as the Traditional Birth Attendants who supervise deliveries at the grassroots including the homes. The 'warm chain' includes creating a warm delivery room, immediate drying, warm resuscitation, warm transportation, skin-to-skin contact with the mother, breastfeeding, postponement of bathing, appropriate clothing and bedding, rooming-in and bedding-in.

It is remarkable that CFR was high among hypothermic babies with severe respiratory distress, sepsis, preterm and asphyxia. Although, the exact cause of death was not confirmed with autopsy, it is attractive to postulate that when these illnesses which are ordinarily associated with high mortality rates [4] are combined with hypothermia, the risk of death is exaggerated. Thus, babies with these diagnoses in addition to hypothermic need specialist attention particularly with respect to thermoregulatory care.

## Conclusion

Hypothermia remains an important clinical finding in neonatal care in a resource-poor setting like Nigeria. The 'warm chain' should be strengthened in all health facilities which offer delivery services and should be incorporated into home-based care of high-risk newborn babies. The skin-to-skin contact method of thermal management, [18] which is appropriate technology, should be encouraged at all levels of health care delivery particularly in resource-poor settings where highly sophisticated warming devices are not usually available in order to reduce the occurrence of neonatal hypothermia and the deaths associated with it.

## Competing interests

The authors declare that they have no competing interests.

## Authors' contributions

TAO conceived, designed and co-ordinated the study and wrote the manuscript. FAA and BMF performed the statistical analysis and interpretation of the data. OBO participated in the design of the study and writing the manuscript while DMO provided critical revision of the manuscript for important intellectual content. All the authors read and approved the manuscript.

## Pre-publication history

The pre-publication history for this paper can be accessed here:


